# Forecast Possible Risk for COVID-19 Epidemic Dissemination under Current Control Strategies in Japan

**DOI:** 10.3390/ijerph17113872

**Published:** 2020-05-29

**Authors:** Zhongxiang Chen, Jun Yang, Binxiang Dai

**Affiliations:** 1 College of Engineering and Design, Hunan Normal University, Yuelu District, Changsha 410081, China; chenzx@hunnu.edu.cn (Z.C.); yangjun@hunnu.edu.cn (J.Y.); 2 School of Mathematics and Statistics, Central South University, Yuelu District, Changsha 410081, China

**Keywords:** COVID-19, SEIHR epidemic model, basic reproduction number

## Abstract

COVID-19 has globally spread to over 4 million people and the epidemic situation in Japan is very serious. The purpose of this research was to assess the risk of COVID-19 epidemic dissemination in Japan by estimating the current state of epidemic dissemination and providing some epidemic prevention and control recommendations. Firstly, the period from 6 January to 31 March 2020 was divided into four stages and the relevant parameters were estimated according to the imported cases in Japan. The basic reproduction number of the current stage is 1.954 (95% confidence interval (CI) 1.851–2.025), which means COVID-19 will spread quickly, and the self-healing rate of Japanese is about 0.495 (95% CI 0.437–0.506), with small variations in the four stages. Secondly, the results were applied to the actual reported cases from 1 to 5 April 2020, verifying the reliability of the estimated data using the accumulated reported cases located within the 95% confidence interval and the relative error of forecast data of five days being less than 2.5%. Thirdly, considering the medical resources in Japan, the times the epidemic beds and ventilators become fully occupied are predicted as 5 and 15 May 2020, respectively. Keeping with the current situation, the final death toll in Japan may reach into the millions. Finally, based on experience with COVID-19 prevention and control in China, robust measures such as nationwide shutdown, store closures, citizens isolating themselves at home, and increasing PCR testing would quickly and effectively prevent COVID-19 spread.

## 1. Introduction

Since the first COVID-19 case was diagnosed in December 2019, COVID-19 had quickly spread to all Chinese provinces by 28 January 2020 [1]. On 1 April 2020, the World Health Organization (WHO) reported 823,636 confirmed cases and 40,598 deaths globally [2]. Among these countries, the most serious epidemic situations were occurring in the United States, Italy, China, Spain, Germany, France, and Iran, which each exceeded 50,000 infected people. In particular, the number of cases in the United States has grown quickly, with the number of reported cases increasing from 15 to 288,721 over 82 days. Contemporaneously, the first COVID-19 case was reported on 15 February 2020 in Japan and the number of accumulated reported cases was 3858 on 5 April 2020. The undocumented infected individuals will facilitate the rapid dissemination of COVID-19 [3]. Therefore, estimation of current infected cases plays an essential role in controlling epidemic development and will help us to evaluate the strategies that should be implemented to adjust the prevention and control measures for mitigating the spread of COVID-19 in Japan.

From 15 to 30 January 2020, the reported cases in Japan were due to imported cases. For instance, the first reported case was a person from Wuhan on 6 January, who had been experiencing symptoms on 3 January. This means that the COVID-19 starting time in Japan was 6 January due to the first imported case not being diagnosed and isolated. Some imported infected or exposed people were in a state of natural transmission until confirmed. According to news reports, the first 10 imported cases, with their serial number, imported days, the symptom days, and confirmed days, are listed in Table 1.

The predominant route of transmission of COVID-19 is person-to-person and dynamic methods can better reflect epidemic law from the aspect of the disease transmission mechanism. To provide theoretical and quantitative bases for making prevention and control decisions, we aimed to establish a mathematical model that reflects the dynamics of COVID-19 propagation, and to qualitatively and quantitatively analyze and numerically simulate the dynamics of the model to show the development process of the disease, reveal the epidemic law, predict the development trend, and analyze the causes and key factors of the epidemic.

Recently, Toshkazu [5] predicted the epidemic peak of COVID-19 in Japan based on a susceptible–exposed–infected–recovered (SEIR) model. This work forecasted that the epidemic peak would be reached in the early to mid-summer and the peak number of confirmed cases in a single day would be $2.053\times 10^{6}$. The works [6,7] on the Diamond Princess cruise ship helped officials to make strategic decisions to prevent COVID-19 from spreading from the ship. These works have a limited impact on future disease prevention and control in Japan and cannot directly explain the key tasks of disease prevention and control. The current state estimation generated by the right model can be useful to govern the epidemic battle in Japan.

In this paper, we establish a seven-compartment dynamic model, and adopt the piecewise method and nonlinear least squares to obtain the parameters in the epidemic model. According to the calculation results, we analyze the key factors that affect the COVID-19 outbreak in Japan. Through numerical examples, we forecast the time when medical resources are scarce and the number of deaths without improving the current situation.

## 2. Methods

### 2.1. Model

**Figure 1:**
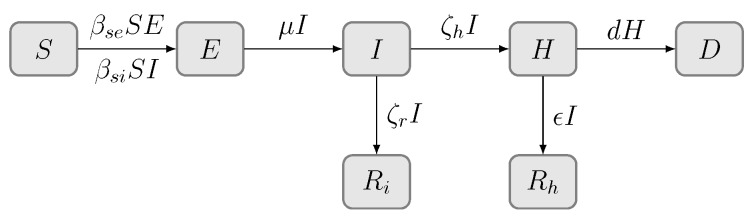
Transmission diagram for the COVID-19 model.

We divided the total human population into seven compartments, as shown in Figure 1, named the SEIHRD model: the susceptible (*S*), the exposed (*E*), the infected (*I*), the hospitalized (*H*), the recovered by self-healing ($R_{i}$), the recovered by hospital curing ($R_{h}$), and the deaths (*D*). The mathematical model is given by:

(1) $$\left\{\begin{array}{ccc} \dot{S}\left(t\right) & = & -\beta_{se}S\left(t\right)E\left(t\right)-\beta_{si}S\left(t\right)I\left(t\right)\\ \dot{E}\left(t\right) & = & \beta_{se}S\left(t\right)E\left(t\right)+\beta_{si}S\left(t\right)I\left(t\right)-\mu E\left(t\right)\\ \dot{I}\left(t\right) & = & \mu E\left(t\right)-\zeta_{r}I\left(t\right)-\zeta_{h}I\left(t\right)\\ \dot{H}\left(t\right) & = & \zeta_{h}I\left(t\right)-\epsilon H\left(t\right)-dH\left(t\right)\\ {\dot{R}}_{i}\left(t\right) & = & \zeta_{r}I\left(t\right)\\ {\dot{R}}_{h}\left(t\right) & = & \epsilon H(t)\\ \dot{D}\left(t\right) & = & dH(t) \end{array}\right.$$

where S(t), E(t), I(t), H(t), $R_{i}\left(t\right)$, $R_{h}\left(t\right)$, and D(t) denote the proportion of susceptible, exposed, infected, hospitalized, removed by hospital cure, removed by self-healing, and deaths in the total populations at time *t*, respectively. Thus, the following equation holds:

(2) $$S\left(t\right)+E\left(t\right)+I\left(t\right)+H\left(t\right)+R_{i}\left(t\right)+R_{h}\left(t\right)+D\left(t\right)=1.$$

where $\beta_{se}$ and $\beta_{si}$ denote the transmission rates from the exposed and infected, respectively. Because COVID-19 is an infectious person-to-person virus, the growth of exposed and infected cases increases the transmission rates $\beta_{se}$ and $\beta_{si}$. Therefore, effective isolation measures and medical screening with the polymerase chain reaction (PCR) test can reduce $\beta_{se}$ and $\beta_{si}$. The exposed individuals become infected after incubation period 1/μ. In reality, a proportion $\zeta_{r}$ recovers from infection through self-cure; $\zeta_{h}$ is the confirmed rate from an infected individual. The hospital cure recovery rate is ϵ and *d* is the proportion of deaths.

**Figure 2:**
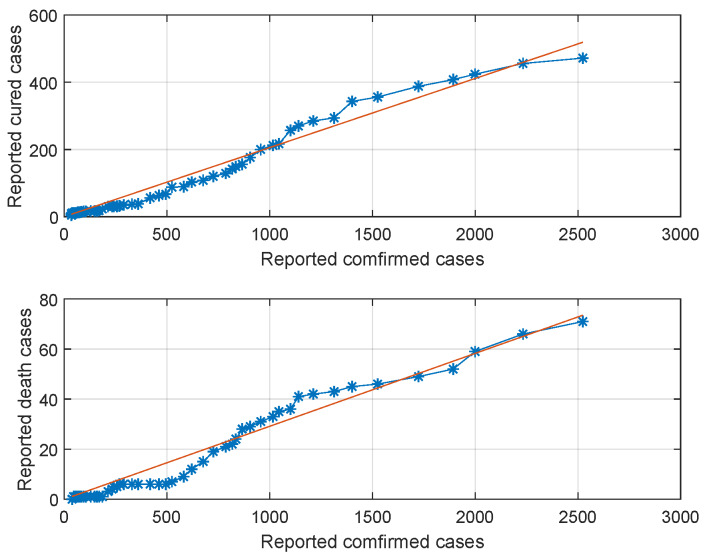
The reported cured and death cases of the reported confirmed cases.

Based on previous research [8,9], 1/μ=5, and hence μ=0.2. In [10], $\beta_{se}=\kappa \beta_{si}$ where κ=0.2. We assume 0.2≤κ≤0.4 because the transmission rate of infected individual is more powerful than that of exposed individuals. Due to the different national fitness and medical conditions of different countries, the hospital cure rate and disease-related mortality rate are also different. According to reported data in Figure 2, we estimated the cure and death rates as ϵ=0.2057 (95% confidence interval (CI), 0.1987–0.2127) and d=0.0291 (95% CI 0.0279–0.0304), as exhibited in Figure 2.

We assumed that the total population is $N=1.26\times 10^{8}$ in Japan [5]. The basic reproduction number $R_{0}$, which is the expected number of secondary cases produced by one infected individual [11], is described as:

(3) $$R_{0}=\frac{\beta_{si}}{\mu }+\frac{\beta_{se}}{\zeta_{h}+\zeta_{r}}.$$

The basic reproduction number $R_{0}$ is independent of the hospital cured rate ϵ and the COVID-19-related death rate *d*. From Equation (1), the number $N\times (H\left(t\right)+R_{i}\left(t\right)+D\left(t\right))$ is the accumulated reported cases, which can be called the documented cases at time *t*. N×I(t) is the undocumented infection cases at time *t*.

### 2.2. Assumption about the Imported Individuals

We used data from the Ministry of Health, Labor, and Welfare, which reports the data related to COVID-19 in Japan [2]. We also collected reported cases from news reports containing more details to guarantee the validity of the data. In Table 1, COVID-19 spread started in Japan on 6 January 2020, which was set as t=0. We assumed that No. 1, No. 2, and No. 7 are the unconfirmed imported infected individuals, and that Nos. 3–6 and 8–10 are imported exposed individuals. We assumed the uniform distribution of the 45 imported exposed individuals that landed from the Diamond Princess cruise ship from 19 to 26 February [3]. Thus, the states of E(t) and I(t) should be updated by the following equations:

(4) $$E\left(t\right)=E\left(t\right)+\bar{E}\left(t\right)$$

and

(5) $$I\left(t\right)=I\left(t\right)+\bar{I}\left(t\right),$$

where $\bar{E}\left(t\right)$ and $\bar{S}\left(t\right)$ indicate the imported proportion at time *t*.

Because the spread of COVID-19 is affected by many factors, different interventions produce different results. Therefore, we estimated the parameters of the model for different stages of the epidemic. We divided the period from 6 January to 31 March into four stages to estimate the model parameters. The first dividing point is 26 February [12], the second dividing point is the implementation of the school suspension order on 6 March [13], and the third is the landing of the Diamond Princess.

### 2.3. Estimation of the Parameters $\beta_{se}$, $\beta_{si}$, $\zeta_{h}$, and $\zeta_{r}$

COVID-19 is a new type of coronavirus, and its detection methods and diagnostic techniques are limited, thus we could not guarantee the integrity of the documented data. Thus, the nonlinear least square method was used to estimate the parameters, and random simulations under isometric observations were used to obtain the confidence intervals of the parameters. We appointed y(t),t=0,1,⋯,85 as the accumulated reported cases of COVID-19 in Japan from 6 January to 31 March 2020. We solved the following least-square-based optimization problem:

For given parameters $\beta_{se}$, $\beta_{si}$, $\zeta_{h}$ and $\zeta_{r}$, the numerical value of Y(t) is:

(6) $$Y(t)=(H(t)+D(t)+R_{h}(t))\times N$$

by employing the model in Equation (1) and the assumption in Section 2.2. We constructed the objective function as:

(7) $$J\left(\beta_{se},\beta_{si},\zeta_{h},\zeta_{r}\right)=\sum {\left[y(t)-Y(t)\right]}^{2}.$$

Then, based on the previous statement, the optimization problem is given by:

(8) $$\min J(\beta_{se},\beta_{si},\zeta_{h},\zeta_{r})$$

subject to:

(9) $$0.2\beta_{si}\leq \beta_{se}\leq 0.4\beta_{si}$$

and:

(10) $$0<\beta_{se},\beta_{si},\zeta_{h},\zeta_{r}<1.$$

**Figure 3:**
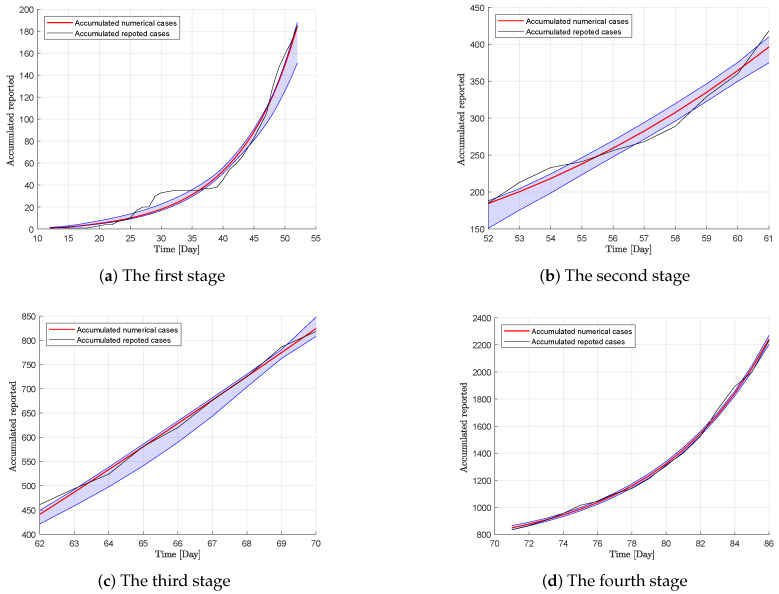
The accumulated reported cases and numerical values with 95% confidence intervals.

By solving the above nonlinear optimization problem employing the lsqnonlin toolbox in MATLAB (MathWorks, Natick, MA, USA) [14], the parameters $\beta_{se}$, $\beta_{si}$, $\zeta_{h}$, and $\zeta_{r}$ can be determined. However, the approximations cannot reflect the error ranges, and the estimation intervals need to be given to confirm that the true values of the unknown parameters are included in these intervals. Based on the bootstrap method [15], we constructed isometric random observations as:

(11) $$\tilde{Y}\left(t\right)=Y\left(t\right)+\chi \left(t\right)$$

where χ(t) denotes random variables from a normal distribution with a mean of zero and variance $\sigma_{i}^{2}$ in the ith stage. Y(t) and χ(t) are calculated by applying $\beta_{se}$, $\beta_{si}$, $\zeta_{h}$, and $\zeta_{r}$. After 1000 simulations, we obtained the calculation results shown in Figure 3 and Table 2. $R_{0}$ was calculated according to Equation (3). The root-mean-square error (RMSE) is 12.264 and the goodness of fit (GOF) $R^{2}$ is 0.9994 from 6 January to 31 March.

## 3. Results

### 3.1. Parameter Explanations

As assumed in Section 2.2, the first dividing point was 26 February and the people disembarked from the Diamond Princess cruise ship were required to isolate for 14 days. This aroused public attention and strengthened self-protection awareness. The transmission rates $\beta_{se}$ and $\beta_{si}$ slightly decreased from the first to the second stage. The second dividing point was the implementation of the decree suspending school and provision of financial assistance to parents. The transmission rates $\beta_{se}$ and $\beta_{si}$ dramatically decreased and the basic reproduction number was close to 1. This measure strengthened the isolation measures and effectively prevented the spread of COVID-19.

After the third dividing point, the transmission rates and confirmed rate substantially changed. During the nine days of the third stage, the number of PCR tests and documented cases were 5550 and 397, respectively. During the 16 days of the last stage, the number of PCR tests and documented cases were 19,471 and 1474, receptively. We defined an average index Υ for the daily PCR tests as:

(12) $$\Upsilon =\frac{\Delta N_{pcr}}{\Delta H\times T}$$

where $\Delta N_{pcr}$, ΔH, and *T* denote the number of PCR tests, the number of documented cases, and the days of the different stages, respectively. We calculated the values of Υ in the last two stages as 1.553 and 0.826, respectively, which showed the confirmed rate decreased sharply and resulted in the growth of transmission rates.

The self-healing rate is within [0.479,0.495] with small variations, showing that this parameter relies on the national constitution. From the trend of the basic reproduction number $R_{0}$ in Table 2, direct and indirect isolation and PCR medical screening measures are effective prevention and control strategies.

### 3.2. Method Examination

**Figure 4:**
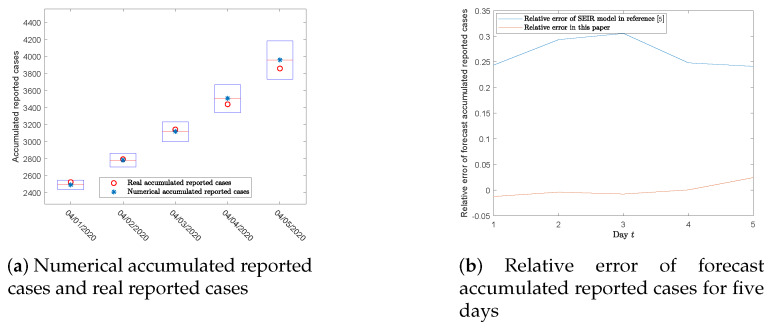
The numerical cases for accumulated reported cases for five days.

With the development of the epidemic, the implemented prevention and control strategies are changing daily. We applied the accumulated reported cases of five days to examine the reliability of our approach. Employing the model in Equation (1) and the parameters in Table 2, the numerical accumulated reported cases and their confidence intervals are 2493 (95% CI 2437–2548), 2780 (95% CI 2700–2860), 3116 (95% CI 2999–3232), 3505 (95% CI 3342–3667), and 3957 (95% CI 3734–4181). All real reported cases exist within the 95% confidence intervals, as proven in Figure 4a.

As demonstrated in Figure 4b, the largest relative error of the five-day forecast data is less than 2.5%, which means the forecast data have high reliability under the current measures. In comparison with the results in [5], the relative errors of forecast data are around 25%, indicating the model and method in this paper are closer to the actual situation.

### 3.3. Predicting the Future Medical State and Deaths Due to COVID-19

To show that the current control strategies may lead Japan to a massive crisis, we combined the existing epidemic bed statistics and the number of ventilators to predict the future medical state. According to a previous investigation [16], the number of existing epidemic beds is about 272,255 and we assumed 45% of the beds are used for other epidemic cases. The number of all kinds of ventilators on standby is about 18,322 including 1255 ECMO devices. According to the real data, we estimated that the proportion of severe cases of COVID-19 in Japan is 5% of the reported cases [16]. When the use of ventilators and/or epidemic beds is saturated, the mortality rate will increase dramatically. Consequently, we estimated the ventilator and epidemic bed saturation times.

**Figure 5:**
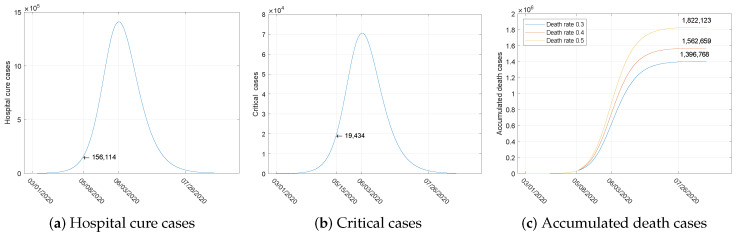
Forecasts of the number of hospital cure cases, critical cases, and accumulated death cases.

As estimated in Figure 5a,b, the hospital cases would reach 156,114, with epidemic beds being insufficient on 8 May and the respirator shortage occurring on 15 May, with about 19,434 critical patients. The results show that makeshift hospitals and shelter hospitals should be arranged in late April at the latest. If we continue with the current situation and adjust the death rate to 3%, 4%, and 5% after the saturation, the number of deaths, as exhibited in Figure 5c, would reach $1.4\times 10^{6}$, $1.6\times 10^{6}$, and $1.8\times 10^{6}$, respectively. To avoid this serious situation, some prevention and control measures that could change the current situation were simulated to predict the epidemic trend.

### 3.4. Effective Intervention Strategies

In this section, we discuss some effective strategies for mitigating COVID-19 dissemination. From Equation (3), some parameters affect the value of the basic reproduction number, such as $\beta_{se}$, $\beta_{si}$, and $\zeta_{h}$. The parameters μ and $\zeta_{r}$ are subject to the characteristics of COVID-19 and the national physique. Hence, we can adopt some appropriate control strategies to impact the transmission rate and the confirmed rate via $\beta_{se}$, $\beta_{si}$, and $\zeta_{h}$. Given China’s experience, the implementation of nationwide shutdowns can effectively reduce the values of $\beta_{se}$ and $\beta_{si}$. Increasing the number of PCR tests and enhancing the investigation of those with direct or indirect contact history can quickly increase the percentage of diagnoses $\zeta_{h}$.

**Figure 6:**
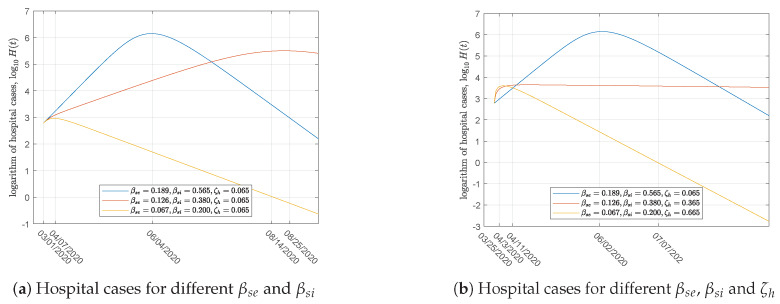
Simulation results using different parameters.

We assumed that nationwide shutdown, store closures, and citizens isolating themselves for at least 28 days are implemented, and that the $\beta_{si}$ decreased to 0.2 starting from March 31. The numerical result exhibited in Figure 6a shows that the peak value decreases and the peak is delayed to 25 August. With the parameters set to $\beta_{se}=0.067$ and $\beta_{si}=0.2$, COVID-19 would be extinct on 14 August in Japan. The above measures appear to be valid strategies for restricting COVID-19 dissemination in Japan.

Essentially, the transmission rate $\beta_{si}$ is decreased when the confirmed rate $\zeta_{h}$ increases. Therefore, in the second numerical example, we changed the parameters $\beta_{se}$, $\beta_{si}$, and $\zeta_{h}$ simultaneously. Figure 6b shows that this effectively shortens the peak time and reduces the peak size, resulting in the earliest COVID-19 virus extinction. Compared with the first simulation, the end of the epidemic would occur on 7 July with $\beta_{se}$, $\beta_{si}=0.067$, and $\zeta_{h}=0.665$, whereas the limited value $\zeta_{h}$ in China is 62% [16].

## 4. Discussion

We applied the SEIHRD model to analyze the development of the COVID-19 epidemic situation in Japan. To eliminate the impact of the uncertainty in the early data, the first 10 imported cases were extracted based on news reports. Table 1 details the days the 10 cases appeared in Japan as infected or exposed individuals. The parameters of the SEIHRD model were estimated by dividing the period from 6 January to 31 March into four segments, and the basic regeneration number $R_{0}$ in the first segment was estimated as 1.614 (95% CI 1.449–1.649), as illustrated in Table 2. On 26 January, due to the landing of the Diamond Princess cruise ship, the officials began to pay attention to the epidemic and introduced relevant isolation measures, which reduced the infection rate and the basic reproduction number $R_{0}$ to 1.484 (95% CI 0.926–1.860). With Japan’s large-scale suspension of schooling on 6 March, the transmission rates weakened to $\beta_{se}=0.105$ (95% CI 0.088–0.202) and $\beta_{si}=0.325$ (95% CI 0.088–0.202). The basic number of $R_{0}$ became 1.053 (95% CI 0.885–1.641). We constructed an index of PCR testing and the proportion of diagnoses $\zeta_{h}$ degraded to 0.065 (95% CI 0.035–0.199) when the index reduced from 1.553 to 0.826. The transmission rates $\beta_{se}$ and $\beta_{si}$ increased as the index decreased. These results show that strengthening the isolation measures and increasing PCR medical screening would help to effectively mitigate COVID-19 spread.

To examine the validity of our approach, applying the SEIHRD model, we generated five-day forecast data and compared the results with the actual accumulated reported cases. The five days of accumulated reported cases exist within the 95% confidence intervals, as shown in Figure 4a. The five-day relative error of the forecasted accumulated reported cases is less than 2.5%, which means this SEIHRD model and the piecewise estimation method can be effective applied to COVID-19. According to the existing medical resources [17], we predicted the time of medical saturation under the current situation and forecast the number of deaths, as shown in Figure 5a.

Numerical examples proved that the suspension of production and business, school shut downs, and large-scale implementation of PCR testing for individuals who may have directly or indirectly come into contact with confirmed cases are the success strategies for preventing the further spread of COVID-19. It may be possible to end the epidemic in early or mid-July by combining isolation measures and increasing PCR testing, as shown in Figure 5b.

In conclusion, there is the possibility of a large-scale outbreak of COVID-19 in Japan given the current situation. Some control strategies such as strengthening the isolation measures and increasing PCR medical screening should be taken to prevent the COVID-19 further spreading, as shown in Section 3.

## Figures and Tables

**Table 1:** First 10 COVID-19 cases imported in Japan [4]. Date format: MM/DD/YYYY.

No.	Imported Day	Sympt. Day	Reported Day	No.	Imported Day	Sympt. Day	Reported Day
1	6 January 2020	3 January 2020	15 January 2020	6	20 January 2020	22 January 2020	28 January 2020
2	19 January 2020	14 January 2020	24 January 2020	7	21 January 2020	21 January 2020	28 January 2020
3	18 January 2020	21 January 2020	25 January 2020	8	12 January 2020	26 January 2020	28 January 2020
4	22 January 2020	23 January 2020	26 January 2020	9	13 January 2020	25 January 2020	30 January 2020
5	12 January 2020	22 January 2020	28 January 2020	10	22 January 2020	23 January 2020	30 January 2020

**Table 2:** Parameter values with 95% confidence intervals for the model in Equation (1).

Parameter	1 January–26 February	27 February–6 March	6–15 March	15–31 March
$\beta_{se}$	0.152(0.145,0.153)	0.145(0.098,0.178)	0.105(0.088,0.202)	0.189(0.182,0.198)
$\beta_{si}$	0.529(0.508,0.532)	0.443(0.304,0.542)	0.325(0.276,0.629)	0.565(0.538,0.597)
$\zeta_{h}$	0.136(0.115,0.257)	0.103(0.075,0.462)	0.136(0.072,0.686)	0.065(0.035,0.199)
$\zeta_{r}$	0.484(0.449,0.490)	0.479(0.376,0.492)	0.479(0.307,0.492)	0.495(0.437,0.506)
$R_{0}$	1.614(1.449,1.649)	1.484(0.926,1.860)	1.053(0.885,1.641)	1.954(1.851,2.025)
